# The Propositional Evaluation Paradigm: Indirect Assessment of Personal Beliefs and Attitudes

**DOI:** 10.3389/fpsyg.2019.02385

**Published:** 2019-11-07

**Authors:** Florian Müller, Klaus Rothermund

**Affiliations:** ^1^Department for the Psychology of Human Movement and Sport, Institute for Sport Science, Friedrich Schiller University, Jena, Germany; ^2^Department for General Psychology II, Institute of Psychology, Friedrich Schiller University, Jena, Germany

**Keywords:** implicit measures, attitudes, propositional beliefs, racism, PEP

## Abstract

Identification of propositions as the core of attitudes and beliefs (De Houwer, 2014) has resulted in the development of implicit measures targeting personal evaluations of complex sentences (e.g., the IRAP or the RRT). Whereas their utility is uncontested, these paradigms are subject to limitations inherent in their block-based design, such as allowing assessment of only a single belief at a time. We introduce the Propositional Evaluation Paradigm (PEP) for assessment of multiple propositional beliefs within a single experimental block. Two experiments provide first evidence for the PEP’s validity. In Experiment 1, endorsement of racist beliefs measured with the PEP was related to criterion variables such as explicit racism assessed *via* questionnaire and indicators of behavioral tendencies. Experiment 2 indicates that the PEP’s implicit racism scores may predict actual behavior over and above explicit, self-report measures. Finally, Experiment 3 tested the PEP’s applicability in the domain of hiring discrimination. Whereas general PEP-based gender stereotypes were not related to hiring bias, results suggest a possible role of female stereotypes in hiring discrimination. In the context of these findings, we discuss both the potential and possible challenges in adopting the PEP to different beliefs. In sum, these initial findings suggest that the PEP may offer researchers a reliable and easily administrable option for the indirect assessment of propositional evaluations.

The desire to assess individuals’ beliefs and attitudes beyond the limits of self-report measures has resulted in indirect measures becoming a staple in psychologists’ toolbox. By tapping into participants’ spontaneous, automatic reactions (i.e., under conditions of reduced intention, control, or awareness concerning the measured construct; see Moors and De Houwer, 2012), they are thought to be less influenced by social desirability or self-presentation and are not subject to the limits of introspection.

Whether and to what extent these measures are actually implicit is the subject of an ongoing debate (Gawronski and De Houwer, 2014)^1^. Recently, however, another limitation of most established indirect measures has attracted attention. As has been pointed out by Hughes et al. (2011), these measures typically attempt to measure associations between concepts (cf. De Houwer et al., 2015). However, this results in *propositional blindness* of these measures, as they allow no distinction based on the specific quality of the relation linking the concepts in question. To give an example, both the statements “I want to be thin” and “I am thin” associate the concepts “I” and “thin.” Both statements differ substantially in meaning, yet this difference cannot be captured by traditional indirect measures that focus on mere associations.

In the following, we briefly characterize two established paradigms that were developed to indirectly assess more complex personal beliefs, the Implicit Relational Assessment Procedure (IRAP) and the Relational Responding Task (RRT). We then introduce the rationale of the Propositional Evaluation Paradigm (PEP) – a sentence priming task in which the evaluation of the task-irrelevant sentence facilitates or interferes with responding in a target categorization task.

## Indirect Measures Targeting Propositions

### Implicit Relational Assessment Procedure

The IRAP (Barnes-Holmes et al., 2010) has been spearheading the development of measures targeting the implicit evaluation of propositions by making the propositional relation between concepts a core feature of its design. For example, participants are shown the phrase “I am” (vs. “I am not”) paired with different positive (vs. negative) adjectives in a series of trials. In one block, they are to respond “correct” to propositions indicative of positive beliefs about themselves (i.e., to propositions combining either the phrase “I am” with a positive attribute or the phrase “I am not” with a negative attribute), whereas they are to respond “correct” to propositions indicative of negative beliefs about themselves in a second block. The performance difference between both types of blocks serves as an index of endorsement of positive relative to negative beliefs about oneself. Although several studies attest to the validity of the IRAP in assessing propositional beliefs (Barnes-Holmes et al., 2010; Hughes and Barnes-Holmes, 2013; Remue et al., 2013, 2014), its practical utility is nevertheless limited by high attrition rates, possibly due to the fact that response key labeling varies on a trial by trial basis.

### Relational Responding Task

To improve on these aspects, De Houwer et al. (2015) developed the Relational Responding Task (RRT). To assess participants’ endorsement of a given belief, a number of sentences are shown that either affirm or contradict this very belief. Participants’ task is to classify these sentences as true or false via button press. Most importantly, in a first block, participants are instructed to perform this classification *as if they would endorse a given belief.* In contrast, they are told to respond in the opposite fashion in a second block (i.e., as if they endorsed the opposite belief). In their study, De Houwer et al. (2015) focused on the belief that Flemish people are more (less) intelligent than immigrants (the study was run in Belgium with Flemish participants). The material therefore consisted of a set of sentences either affirming (e.g., “Flemish people are smarter than immigrants”) or contradicting this belief (e.g., “Flemish people are dumber than immigrants”). In a first block, participants were to respond *as if they held the belief* that Flemish people were in fact smarter than immigrants. In contrast, they were to respond *as if they held the opposite belief* in a second block. On selected trials, no sentence was presented and participants had to react to synonyms of “true” or “false” (e.g., correct, valid, incorrect, invalid) by pressing the corresponding key (De Houwer et al., 2015, p. 4). These additional “response label trials” (Eder and Rothermund, 2008) were introduced in order to prevent recoding of the response keys (i.e., participants might otherwise treat the “false” key as a “true” response and vice versa in the block that requires them to assume a counter-attitudinal stance, allowing them to respond on the basis of their true attitudes).

Highlighting the RRT’s potential to assess individual differences in propositional evaluation, RRT scores correlated with explicit measures assessing participants’ beliefs regarding immigrants (subtle, blatant, and modern racism scales; McConahay, 1986; Pettigrew and Meertens, 1995). In line with the goal to reduce the task’s demands on participants, both task duration and attrition rate were substantially lower than those observed in the IRAP (De Houwer et al., 2015, p. 6).

However, by inheriting the block structure from the IRAP, the RRT is also subject to limitations that are inexorably tied to this design. First and foremost, like in the IRAP, personal evaluations of one and only one belief can be assessed in a single RRT. This follows from the requirement of having to instruct participants for each block on the basis of which specific attitudinal stance they are to respond. For example, participants are instructed to respond as if they believe that immigrants are less (or more) intelligent than the host population. Thus it is impossible to assess personal evaluations of additional beliefs within the same task, because this would require additional instructions that would have to be applied simultaneously in the same block, rendering the task ambiguous. Second, the reaction time difference between both blocks is seen as indicative of participants’ relative endorsement of the two instructed beliefs. However, other factors that are unrelated to attitudes and beliefs might also be driving block effects. For example, participants might differ in their ability to simulate the perspective required by the current block’s instruction due to differences in cognitive flexibility: the more adept participants are in implementing the instructions, the smaller the resulting block difference – irrespective of actual beliefs (this problem resembles the “cognitive skill confound” that was identified with regard to the dual block procedure of the IAT, McFarland and Crouch, 2002; see also Back et al., 2005; Klauer et al., 2010). Additional arguments have been made regarding method-specific variance driving the block difference in the IAT (Mierke and Klauer, 2003; Teige-Mocigemba et al., 2008; Rothermund et al., 2009); similar concerns might also apply to the RRT and the IRAP.

## Automatic Evaluation in Reading

To both build upon the innovations introduced with the RRT and address the aforementioned drawbacks, we drew inspiration from research on language comprehension investigating the (automatic) evaluation of statements’ validity. (Wiswede et al., 2013; see also Richter et al., 2009; Isberner and Richter, 2013, 2014) employed a sentence priming paradigm that presented statements that were either true or false (e.g., “Milk is white” or “Saturn is not a planet”) in a word by word fashion (Rapid Serial Visual Presentation, RSVP). Most importantly, however, participants’ response did not depend on the sentence primes, which were irrelevant for the task at hand. Instead, they had to respond to the target words “true” or “false” that were presented after the sentence by pressing the corresponding key. Because all sentences were presented with both types of targets, the congruency effect between the required response and the sentences’ validity could be estimated. Results demonstrated that participants’ reaction times were significantly shorter for congruent (responding with “true” [“false”] after a true [false] sentence) compared to incongruent trials.

The paradigm employed by Wiswede et al. (2013) removes the restrictions of the RRT and IRAP that were discussed previously. First, there is no need for instructions on how to evaluate the presented statements, because participants’ reaction is solely dependent on the response prompt. This removes the need for separate blocks and also allows the assessment of participants’ reactions to a diverse set of statements not limited to one specific belief. Finally, because participants do not have to react as if they endorse a given belief, there is no need to conduct the task in separate blocks, eliminating method variance related to the block design (see Mierke and Klauer, 2003; Rothermund et al., 2009, as discussed earlier).

## The Propositional Evaluation Paradigm

We propose the Propositional Evaluation Paradigm (PEP) modeled after Wiswede et al. (2013) as an alternative method to assess evaluation of statements that have no *a priori* truth value. As a priming paradigm, each trial of the PEP consists of a task-irrelevant sentence presented in a word by word fashion (RSVP) to the participant. After a brief interval, the task-relevant target stimulus – either the word “true” or “false” – is presented on screen and participants are to press the corresponding key. To ensure that the prime sentences are attended to, a number of “catch trials” require participants to react according to specific properties of the item (see “Method” section for details). This is indicated by the response prompt “?? false – true ??”.

The extent to which participants tend to evaluate a sentence as true vs. false manifests itself in the difference of the reaction times for the “true” vs. “false” response prompts for a given sentence. In this task, each sentence serves as its own control, which eliminates error variance that relates to differences in participants’ general response speed.

Note that the PEP has been shown to differentiate between simple sentences that are unambiguously true or false (Wiswede et al., 2013). In the current study, we sought to demonstrate that the PEP is also able to capture *individual differences in beliefs*. We therefore conducted a series of three studies that tested the PEP in different contexts. First, we attempted to replicate previous research on the RRT (De Houwer et al., 2015) by using the PEP to predict explicit attitudes and behavioral intentions toward refugees. Second, we broadened the scope by using the PEP to predict actual pro-refugee behavior. In the third and final study, we tested the PEP’s ability to predict differences in behavior in a different context, that is, in the domain of gender-based hiring discrimination.

## Experiment 1

To facilitate comparisons with previous research, this first experiment mirrored the design by De Houwer et al. (2015). Specifically, we assessed individuals’ attitudes concerning refugees with adapted versions of the Classic Racism Scale and the Modern Racism Scale (Akrami et al., 2000). The very same items used in these scales were also used as stimuli in the PEP, which guarantees perfect comparability of both measures of racist attitudes, and allows direct assessment of the PEP’s validity. In addition, participants’ political orientation and behavioral intentions concerning actions in support or against refugees were collected.

### Method

#### Sample

A total of 92 participants^2^ (74% female, Age: *M* = 22.2, SD = 4.75, Range = 18–57) were recruited on campus of the Friedrich Schiller University (Jena, Germany) and compensated with course credit or sweets. An ethics approval was not required as per applicable institutional and national guidelines and regulations because no cover-story or otherwise misleading or suggestive information was conveyed to participants (this procedure is in accordance with the ethical standards at the Institute of Psychology of the University of Jena). Participants indicated their informed consent by agreeing via button press at the beginning of the experiment. Otherwise, the study was terminated at this point (i.e., participants did not continue to the study proper)^3^.

#### Procedure

Upon arrival at the laboratory, participants were seated in individual, sound proof cubicles and received further instructions on screen. Specifically, they learned that they were to complete a reaction time task followed by a set of questionnaires. Participants were encouraged to contact the experimenter should questions arise. Detailed instructions were given immediately before each part of the experiment.

##### Assessment of Racism With the PEP

**Figure 1:**
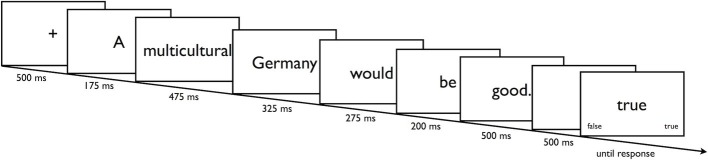
Presentation of an individual item in a PEP trial. Note that presentation time accounts for differences in word length.

In a series of trials, participants were shown all eight items of the Classic Racism Scale and all nine items of the Modern Racism Scale (Akrami et al., 2000). Similar to the procedure by Wiswede et al. (2013), following a fixation cross (500 ms), a specific item was presented in a word by word fashion in the center of the screen (RSVP, see Figure 1). Whether the items were from the Classic or Modern Racism Scale and whether they expressed positive or negative attitudes toward refugees constituted the within-subject factors *Scale* (CR, MR) and *Attitude* (positive, negative). Presentation time accounted for differences in word length by extending the base presentation time of 150 ms by 25 ms for each letter. Thus the word “refugees” would have been presented for 150 ms + (25 ms × 8 letters) = 350 ms. The final word of each item was always presented for 500 ms. After a 500-ms blank interval, the response prompt (the word “true” or “false”) indicated to participants whether to press the corresponding “true” or “false” key. The prompt shown constituted the within-subject factor *Required Response* (true, false). Each item was shown with each response prompt resulting in (8 + 9) × 2 = 34 individually randomized trials. Participants completed three blocks of these trials, resulting in a total of 34 × 3 = 102 experimental trials.

To ensure that participants actually read the sentences (recall that reading the sentence primes is in fact irrelevant for responding correctly to the response prompt), an additional set of 10 sentences were interspersed with the material. These “catch trials” actually had to be evaluated by participants, indicated by a different response prompt: “? false – true ?”. Each of these sentences was shown three times, yielding an additional 10 × 3 = 30 trials. In order to familiarize participants with the upcoming task, a practice block of six trials was administered (materials differed from the stimuli used in the experimental trials).

##### Explicit Assessment of Racism

Following the PEP, both the Classic Racism Scale and Modern Racism Scale – that is the very same items that were presented as sentence primes in the PEP – were administered via questionnaire. For each item, participants indicated their agreement on a 5-point rating scale ranging from 1 = “not at all” to 5 = “absolutely.” Items expressing positive attitudes toward refugees were reversed before averaging the items of each scale to compute separate indices for classic (Cronbach’s *α* = 0.66) and modern racism (Cronbach’s *α* = 0.73).

##### Assessment of Behavioral Indicators

Two items assessed how likely participants were to take action in favor of or against refugees (i.e., “Do you want to get involved with supporting refugees?” and “Do you want to take action against further immigration of refugees?”) on a 5-point rating scale ranging from 1 = “not at all” to 5 = “absolutely.” Ratings on these items were negatively correlated (*r* = −0.31, *p* = 0.005) and therefore combined into one behavioral index (after recoding the negative item). Two additional items assessed whether participants were actually involved in activities in favor of or against refugees (“yes,” “no”) and provided the option to describe these actions (free text). Because only one participant indicated involvement in activities against refugees, this item was dropped from further analyses. Finally, a single item asked participants to indicate their own political orientation on a 10-segment scale ranging from “left” to “right.”

##### Funneled Debriefing

The questionnaire concluded with collecting participants’ comments concerning the reaction time task, their strategies in dealing with the reaction time task, and their suspicions concerning the hypotheses investigated in the current study.

### Results

To reduce the influence of outliers on reaction times, data were prepared as follows. First, trials with incorrect responses (6.64%) as well as global reaction time outliers (i.e., RT < 150 ms; RT > 2,500 ms) were removed (1.1%). Second, reaction times exceeding the mean of an individual’s respective reaction time distribution^4^ by more than two standard deviations (2%) were removed. Exclusion of participants performing at less than 80% accuracy^5^ in the PEP resulted in a final sample size of *N* = 82 (i.e., an attrition rate of 11%).

#### Indirect Measurement of Racism With the PEP

**Figure 2:**
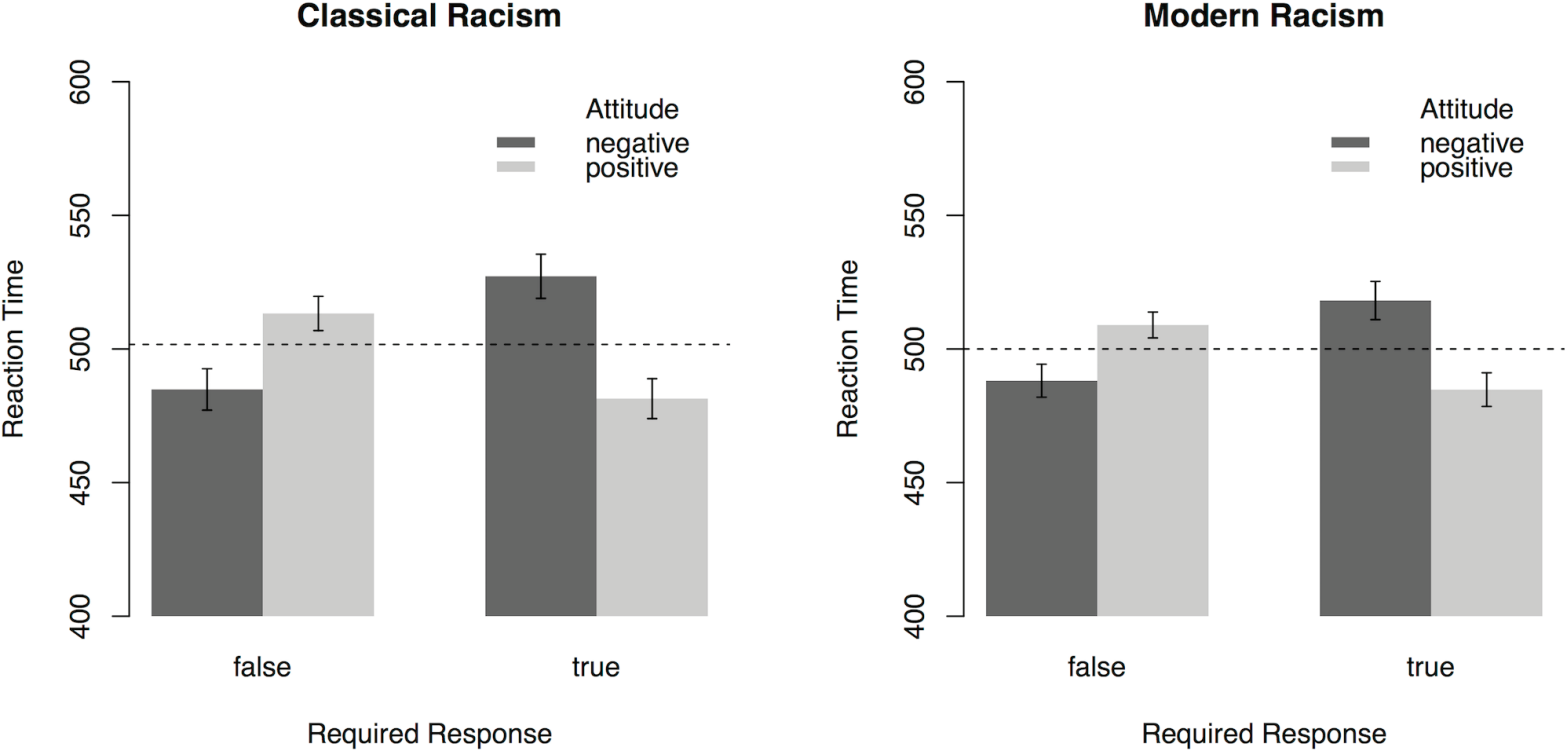
Reaction times (error bars indicate 95% CI) in the PEP depending on *Attitude* toward refugees expressed in the sentences, *Required Response*, and the type of *Scale* (Experiment 1). Dashed lines represent mean reaction time. On the sample level, results indicate that participants associate positive attitudes more strongly with “true” responses, with the reverse being true for negative attitudes.

In a first step, we investigated general trends of spontaneous evaluations for statements expressing either positive or negative attitudes toward refugees. For this purpose, averaged RTs for categorizing the “true”/“false” response prompts after sentence primes were subjected to a 2 (*Attitude:* positive, negative) × 2 (*Required Response:* true, false) × 2 (*Scale:* Classic Racism Scale, Modern Racism Scale) ANOVA with repeated measurement on all factors. A main effect of *Attitude*, *F*(1, 81) = 10.74, *p* = 0.002, $\eta_{p}^{2}$ = 0.12, was qualified by the interaction of *Attitude* × *Required Response*, *F*(1, 81) = 68.55, *p* < 0.001, $\eta_{p}^{2}$ = 0.46. As illustrated in Figure 2, “true” (“false”) targets were categorized faster after sentences expressing positive (negative) attitudes toward refugees, respectively. No other effects were significant (all *p*s > 0.06). This analysis demonstrates that participants’ reaction times vary depending on the attitudes expressed in the item and the response required by the response prompt. Faster responses for “true” targets after statements expressing positive attitudes and faster responses for “false” targets after statements expressing negative attitudes indicate an overall endorsement of positive attitudes toward refugees.

#### Predicting Explicit Racism and Behavioral Intentions

In order to relate individual differences in these reaction time patterns to differences in questionnaire-based indices of racism, a new variable representing the interaction of *Attitude* × *Required Response* in the ANOVA was computed on the aggregated trials representing each factor combination as follows:


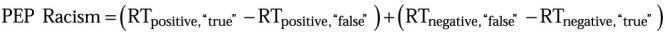


This index of implicit racism was computed separately for each of the two racism scales, with more positive values indicating more pronounced racism, that is, more negative attitudes toward refugees. Scores for Classic Racism correlated highly with Modern Racism irrespective of whether these attitudes were measured via PEP (*r* = 0.39, *p* < 0.001, 95% CI: 0.19–0.56, BF_10_ = 82.37) or questionnaire (*r* = 0.48, *p* < 0.001, 95% CI: 0.30–0.63, BF_10_ = 4376.4). Therefore, scores for Classic and Modern Racism were averaged both for the PEP and for the questionnaire data to form global racism scores.

To validate the PEP as an indirect measure of propositional evaluation, its utility in predicting both explicit, questionnaire-based measures of racism and behavioral intentions indicative of racism was evaluated. Thus, correlations between the racism score from the PEP and these measures were computed. First, racism assessed via PEP correlated with racism assessed via questionnaire (*r* = 0.37, *p* < 0.001, 95% CI: 0.17–0.54, BF_10_ = 43.27). Thus, the PEP is able to assess individual differences in beliefs, similar to questionnaire-based measures. Second, the same pattern of results was observed concerning behavioral intentions, regardless of whether racism was assessed via PEP or questionnaire (see Table 1 for a correlation matrix). Higher racism was related to weaker intentions for pro-refugee behavior (PEP: *r* = −0.33, *p* < 0.01, 95% CI: −0.51 to −0.012, BF_10_ > 12.49; Questionnaire: *r* < −0.70, *p* < 0.001, 95% CI: −0.80 to −0.58, BF_10_ = 46.64E9). The same held for political orientation – higher racism was associated with stronger preferences for the right end of the political spectrum, regardless of whether racism was assessed via PEP or questionnaire (PEP: *r* = 0.26, *p* = 0.02, BF_10_ = 2.06; Questionnaire: *r* = 0.44, *p* < 0.001, BF_10_ = 546.82).

**Table 1:** Correlations between PEP-based racism, questionnaire-based racism (QNR), and various outcomes in Experiment 1 (PO: Political Orientation).

	Racism		Outcomes	
	PEP	QNR	Intentions	PO
PEP	1	0.37**	−0.33**	0.26*
QNR		1	−0.70***	0.44***
Intentions			1	−0.52***

* *p < 0.05*;

** *p < 0.01*;

*** *p < 0.001*.

In order to investigate the incremental validity of the PEP over and above explicit questionnaires, both racism scores were used as predictors in a multiple regression. In predicting behavioral intentions and political orientation, only explicit racism emerged as a significant predictor (all *p*s < 0.001; for implicit racism, all *p*s > 0.32).

#### Reliability of the PEP

Split-half (odd-even) reliability of the PEP score yielded a Spearman-Brown corrected *r* = 0.72. Thus, reliability of the PEP seems to slightly exceed the reliability of the RRT (*r* = 0.64, De Houwer et al., 2015, p. 6).

### Discussion

It was the goal of Experiment 1 to demonstrate that the assessment of attitudes with the PEP is sensitive to individual differences. As expected, racism assessed with the PEP was highly correlated to racism scores from standard questionnaires. Likewise, racism assessed with the PEP was related to the same criterion variables (behavioral intentions, political orientation) as racism assessed via questionnaire.

Of course, the usefulness of a measure that is undeniably more complicated than standard questionnaires needs to provide incremental validity. In the current study, this was not the case as racism assessed with the PEP did not predict criterion variables over and above racism assessed with questionnaires. However, note that the current study used self-reported explicit behavioral intentions relating directly to participants’ attitudes toward refugees as criterion variable. It is not surprising that an indirect measure of propositional evaluations and beliefs does not outperform explicit attitude measures in predicting this outcome. Such deliberative judgments (vs. spontaneous reactions) have been shown to be closely related to explicit self-report measures (see Fazio et al., 1995, p. 1018; Dovidio et al., 1997, p. 512; Pearson et al., 2009, p. 322).

Furthermore, the current version of the PEP featured catch trials ensuring that participants actually read the presented items. Specifically, participants were asked to *actually evaluate* selected items on a number of trials by pressing the appropriate response key. Even though the PEP’s regular “true”/“false” response prompt clearly indicated that an evaluation of the sentence was not required on standard trials, the additional task that had to be applied during the catch trials might have induced participants to transfer the explicit evaluation task to the test trials also – even though such an explicit evaluation was not required. This feature of the task might compromise its classification as a fully implicit measure, in that it is not perfectly goal-independent. We will address the question of automaticity again in the general discussion, after having introduced another version of the PEP that uses a different type of additional task.

## Experiment 2

In order to improve upon the previously discussed aspects, Experiment 2 featured revised catch trials to ensure that participants attend to the presented items without requiring an explicit evaluation of the truth value of the respective sentences during the experiment. Specifically, participants were to indicate on selected trials whether the presented item contained a spelling error. This rendered it unlikely that participants formed an intention to evaluate the truth/falsity of the presented sentences according to their own explicit attitudes, while ensuring that the presented sentences were not ignored.

In addition to the previously employed self-reported behavioral intentions, we included a measure of spontaneous behavior as an outcome variable, because established research has documented a close relationship between indirect measures of racism and spontaneous behavior lacking clear standards for appropriate behavior (Fazio et al., 1995; Dovidio et al., 1997; Pearson et al., 2009). Specifically, participants’ persistence in a color matching task that determined donations supporting refugees served as an indicator of spontaneous pro-refugee behavior.

### Method

#### Sample

A total of 65^6^ participants completed the experiment after having been recruited on campus of the Friedrich Schiller University (Jena, Germany). Exclusion of six participants who either left the experiment prematurely or questioned the meaning of the color matching task resulted in a final sample size of *N* = 59 (63% female, Age: *M* = 21.4, SD = 2.72, Range = 18–33). Participants were compensated with course credit or sweets.

#### Procedure

Upon arrival at the laboratory, participants were seated at individual tables and received further instructions on screen. Specifically, they learned that they were to complete a reaction time task (i.e., the PEP) followed by a set of questionnaires and a final reaction time task (i.e., the color matching task assessing spontaneous pro-refugee behavior). Participants were encouraged to contact the experimenter should questions arise. Detailed instructions were given immediately before each part of the experiment.

##### Assessment of Racism With the PEP

Presentation and timing mirrored the previous study. Again, stimulus sentences were either from the Classic Racism Scale or the Modern Racism Scale expressing positive and negative attitudes toward immigrants, constituting the within-subject factors *Scale* (CR, MR) and *Attitude* (positive, negative). Identical to the previous study, the response prompt indicated the appropriate reaction, constituting the within-subject factor *Required Response* (“true,” “false”).

However, the current experiment differed in the nature of the catch trials. Whereas Experiment 1 required participants to indicate whether they believed a sentence to be either true or false, the response prompt “?? false – true ??” now signaled to evaluate whether the presented sentence contained a spelling error. Participants indicated their response by pressing the “true” or “false” key. Therefore, all sentences existed in two versions, one featuring a spelling error and one without, represented by the within-subjects factor *Spelling* (correct, wrong).

All sentences were shown twice with both the “true” and the “false” prompt, resulting in 2 × (17 sentences × 2 spelling versions × 2 prompts) = 136 trials. Additionally, all sentences were shown twice with the “?? false – true ??” prompt indicating evaluation of spelling, yielding an additional 2 × (17 sentences × 2 spelling versions × 1 prompt) = 68 trials. Thus, participants completed a total of 136 + 68 = 204 trials.

##### Explicit Assessment of Racism

Mirroring the previous experiment, participants then completed the Classic and Modern Racism Scale. Again, items expressing positive attitudes toward refugees were reversed before averaging the items of each scale to compute separate indices for classic (Cronbach’s *α* = 0.76) and modern racism (Cronbach’s *α* = 0.72).

##### Assessment of Behavioral Indicators

As in Experiment 1, two items assessed how likely participants were to take action in favor of or against refugees on a 5-point rating scale ranging from 1 = “not at all” to 5 = “absolutely.” Again, items were negatively correlated (*r* = −0.34, *p* < 0.01), and thus averaged to form one behavioral index (after recoding the negative item). Two items assessed participants’ involvement in activities in favor of or against refugees (“yes,” “no”) and provided the option to describe these actions (free text). Because only one participant indicated involvement in activities against refugees, this measure was dropped from further analyses.

##### Assessment of Spontaneous Behavior

To assess participants’ spontaneous behavior toward refugees, we employed a color matching task modeled after Freund (2006). In each trial, participants were shown a rectangle filled with a random color on screen. Whereas the saturation of the rectangle’s top half fluctuated randomly, participants’ task was to adjust the saturation of the rectangle’s bottom half to match the saturation of the upper half by moving the mouse up or down. After 20 s, a new trial started with a new random fill color. Participants were told that they could exit the task at any time by pressing the escape key. However, they also knew that the better they managed to follow the target saturation and the longer they persisted in the task, the more points they would accumulate. The amount of points accumulated by all participants then determined the amount donated to a local non-profit organization supporting refugees. Thus actual time spent on the task served as a subtle, indirect measure of pro-refugee behavior.

### Results

PEP effects were computed on trials featuring correct spelling. First, trials with incorrect responses (7%) as well as global reaction time outliers (i.e., RT < 150 ms, RT > 2,500 ms) were removed (0.5%). Second, reaction times exceeding the mean of an individual’s respective reaction time distribution^3^ by more than two SDs (3.1%) were removed. Three participants were excluded from the sample because they achieved less than 80% accuracy in the PEP, resulting in a final sample of *N* = 56 (5% attrition rate).

#### Indirect Measurement of Racism With the PEP

**Figure 3:**
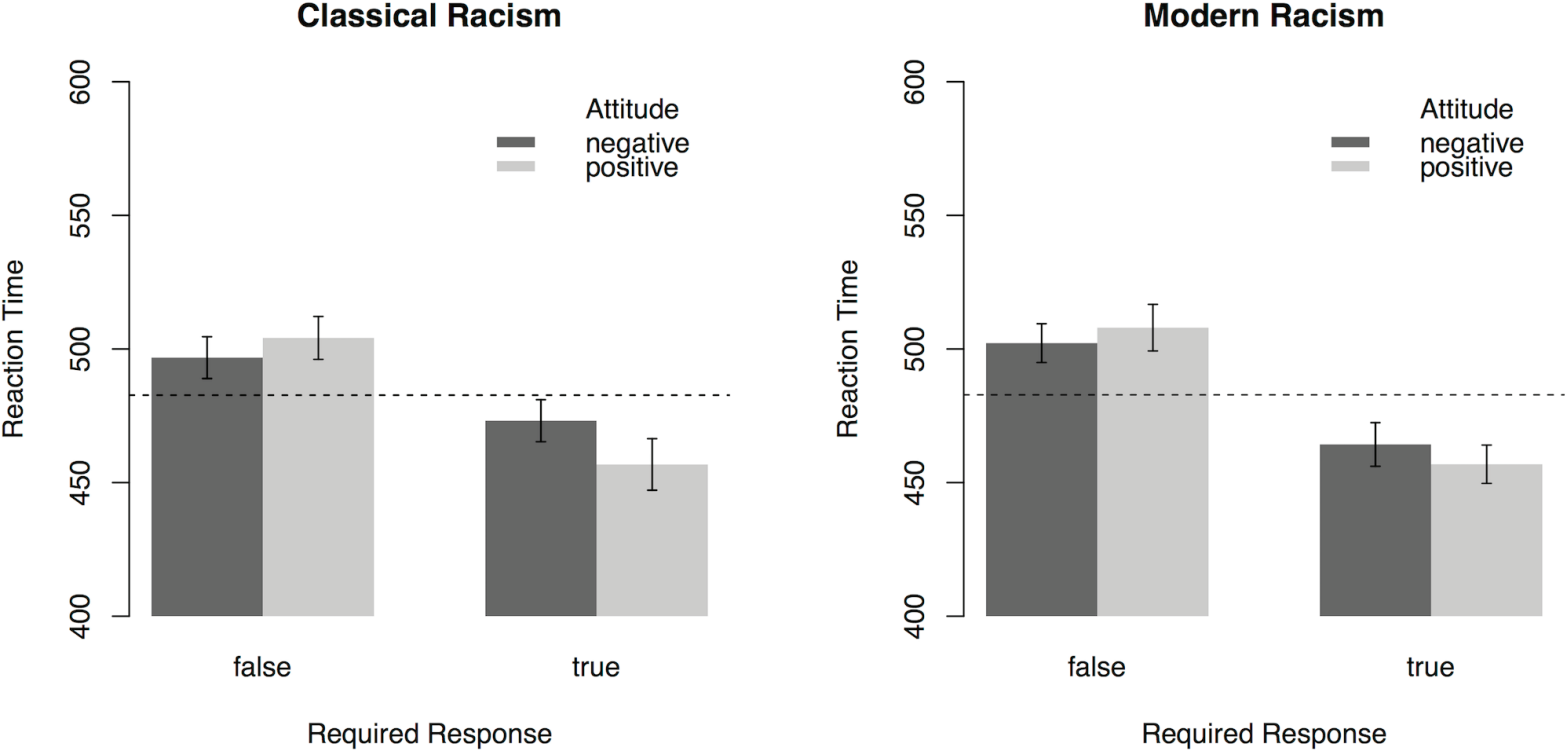
Reaction times (error bars indicate 95% CI) in the PEP depending on *Attitude* toward refugees expressed in the sentences, *Required Response*, and the type of *Scale* (Experiment 2). Dashed lines represent mean reaction time. On the sample level, results indicate that participants associate positive attitudes more strongly with “true” responses, with the reverse being true for negative attitudes.

To analyze general trends in attitudes toward immigrants, participants’ reaction time for categorization of the response prompt following orthographically correct sentences was subjected to a 2 (*Scale*: CR, MR) × 2 (*Attitude*: positive, negative) × 2 (*Required Response*: true, false) ANOVA with repeated measurement on all factors. As illustrated in Figure 3, the interaction effect of *Attitude* × *Required Response*, *F*(1, 55) = 6.7, *p* = 0.01, $\eta_{p}^{2}$ = 0.11, indicated that “true” (“false”) targets were categorized faster after sentences expressing positive (negative) attitudes toward refugees, respectively. Except for a main effect of *Required Response*, *F*(1, 55) = 81.72, *p* < 0.001, $\eta_{p}^{2}$ = 0.60, no other effects were significant (all *p*s > 0.15). Mirroring the results from Experiment 1, the interaction effect demonstrates that participants’ reaction times depend on the attitudes expressed in the item and the required response. Faster responses for “true” targets after statements expressing positive attitudes and faster responses for “false” targets after statements expressing negative attitudes indicate an overall endorsement of positive attitudes toward refugees.

#### PEP Racism and Explicit Racism

Following the procedure in Experiment 1, a new variable representing the interaction effect of *Attitude* × *Required Response* from the ANOVA was computed for each participant, separately for each racism scale. Because participants’ PEP scores for Classic and Modern Racism were not correlated (*r* = 0.14, *p* = 0.31, 95% CI: −0.13 to 0.39, BF_10_ = 0.49, in contrast to questionnaire-based scores, *r* = 0.59, *p* < 0.001, 95% CI: 0.38–0.74, BF_10_ = 9002.33), both scales were analyzed separately. Attesting to the efficacy of the changes in the catch trials compared to Experiment 1, neither classic racism nor modern racism assessed via the PEP were related to their questionnaire-based counterparts (Modern Racism: *r* = −0.08, *p* = 0.55, 95% CI: −0.34 to 0.19, BF_10_ = 0.35; Modern Racism: *r* = 0.09, *p* = 0.51, 95% CI: −0.18 to 0.35, BF_10_ = 0.37).

#### Predicting Self-Reported and Actual Behavior

To analyze the relative influence of PEP and questionnaire-based classic and modern racism on behavioral measures, these four racism scores served as predictors in multiple regressions (see Table 2 for pairwise correlations).

**Table 2:** Correlations between PEP-based racism, questionnaire-based racism (QNR), and various outcomes in Experiment 2 (CR = Classic Racism, MR = Modern Racism).

		PEP		QNR		Outcomes	
		CR	MR	CR	MR	Intentions	Time on task
PEP	CR	1	0.14	−0.08	−0.17	0.06	0.06
	MR		1	−0.11	0.09	0.04	−0.34**
QNR	CR			1	0.59***	−0.61***	−0.20
	MR				1	−0.76***	−0.42**
	Intentions					1	0.36**

** *p < 0.01*;

*** *p < 0.001*.

Concerning behavioral intentions, only the questionnaire-based racism scores emerged as significant predictors such that lower levels of both Classic and Modern Racism were related to more positive behavioral intentions toward refugees (Classic Racism: *p* = 0.037, Modern Racism, *p* < 0.001; see Table 3, top). In contrast, PEP-based measures of racism did not predict behavioral intentions (Classic Racism: *p* = 0.37, Modern Racism, *p* = 0.35; see Table 3, top).

**Table 3:** Multiple regressions for behavioral intentions (top) and time spent on color matching task (bottom) on PEP- and questionnaire-based (QNR) Classic and Modern Racism in Experiment 2 (CR = Classic Racism, MR = Modern Racism).

	Predictor	*B*	SE *B*	*β*	SE *β*	*t*	*p*
**DV: Behavioral intentions**							
QNR	Intercept	6.6	0.29	4.03	0.07	60.36	<0.001***
	CR	−0.31	0.14	−0.18	0.09	−2.14	0.037*
	MR	−0.9	0.15	−0.51	0.09	−5.97	<0.001***
PEP	CR	−9.1E-4	1E-3	−0.06	0.07	−0.91	0.37
	MR	9.3E-4	9.7E-4	0.07	0.07	0.95	0.35
**DV: Time on color matching task**							
	Intercept	839.36	173.57	366.92	40.08	9.15	<0.001***
QNR	CR	−1.97	86.54	−1.17	51.18	−0.02	0.982
	MR	−229.09	90.84	−130.7	51.83	−2.52	0.015*
PEP	CR	0.16	0.60	11.41	41.62	0.27	0.785
	MR	−1.47	0.58	−106.18	42.10	−2.52	0.015*

*t for standardized beta’s*.

* *p < 0.05*;

*** *p < 0.001*.

In contrast, concerning actual behavior as operationalized by the time spent on the color matching task, both higher PEP- and questionnaire-based modern racism yielded less time on task (Questionnaire: *p* = 0.015, PEP: *p* = 0.015; see Table 3, bottom). Most importantly, PEP-based modern racism scores predicted time on task in addition (and to a similar extent) to questionnaire-based modern racism scores. In contrast, classic racism scores did not predict time on task irrespective of type of measurement (Questionnaire: *p* = 0.98, PEP: *p* = 0.79).

#### Reliability of the PEP

Split-half (odd-even) reliability of the PEP score yielded a Spearman-Brown corrected *r* = 0.54 for modern and *r* = 0.31 for classic racism.

### Discussion

Experiment 2 introduced a variant of the PEP that used an additional task during the “catch trials” that does not require an evaluation of the content or truth status of the sentences. Instead, participants only had to judge the orthographical correctness of the presented sentences. Rendering truth evaluation completely task irrelevant during the PEP did not eliminate the compatibility effect, that is, responses were still faster for “true” (“false”) prompts after having read statements expressing pro- (anti-)refugee attitudes. However, correlations of classic and modern racism PEP scores with questionnaire-based classic and modern racism as well as self-reported behavioral intentions were absent for this version of the PEP, which is initial evidence that explicit attitudes may no longer influence responding during the PEP.

Importantly, including a measure of spontaneous behavior (i.e., time spent on color matching task) allowed us to test the hypothesis that implicit endorsement of sentences expressing pro- vs. anti-refugee attitudes *via* the PEP predicts spontaneous behavior over and above explicit attitudes. In line with this prediction, we found that modern racism assessed with the PEP predicted persistence on a task that was linked to pro-refugee outcomes (time spent on the task translated into money sent to a pro-refugee organisation). This relationship was not found for classic racism – irrespective of type of measurement. Whereas this differential pattern of predictive validity for PEP items belonging to the Classic and Modern Racism Scales was not predicted, it is in line with findings by Akrami et al. (2000) reporting higher sensitivity of the modern racism scale for individual differences (due to floor effects of the blatant, classic racism scale).

The first two experiments provide initial evidence that the PEP is able to measure individual differences in attitudes (Experiments 1 and 2) and may contribute to predicting actual behavior over and above explicit, questionnaire-based measures (Experiment 2). However, these findings have been limited in scope to the domain of racial discrimination. To further test the utility of the PEP in predicting behavior, the next experiment adopted the current rationale to the context of stereotype-driven hiring discrimination.

## Experiment 3

The role of stereotypes in biasing hiring decisions has been documented in various domains, such as weight (Agerström and Rooth, 2011), age (Diekman and Hirnisey, 2007), and gender (Eagly and Karau, 2002). One explanation for these effects is provided by Role Congruity Theory (Eagly and Karau, 2002) positing that the stereotypical traits of a social group and a given job’s requirements may be more or less aligned (i.e., congruent). For example, to the extent that old people are stereotypically seen as less flexible and open for change, they will be less likely to be hired in a position thought to demand this very flexibility (Diekman and Hirnisey, 2007). In the case of gender bias, Eagly and Karau (2002) argue that hiring biases against women in leadership positions may result from the incongruence of stereotypically female traits (e.g., communal attributes; Bakan, 1966) and the stereotypically male qualities (e.g., agentic attributes) associated with leadership (see also Rudman and Glick, 2001).

Building upon these findings, we used both the PEP and a questionnaire to assess individuals’ gender stereotypes, i.e., the extent to which agentic and communal attributes were differentially associated with both sexes. We then asked participants to select among male and female applicants those who they believed to be best suited for a number of job descriptions emphasizing either agentic (male) or communal (female) requirements.

### Method

#### Sample

A total of *N* = 48^7^ (65% female, Age, *M* = 23.5, SD = 4.01, Range = 18–38) participants were recruited on campus of the Friedrich Schiller University (Jena, Germany), and compensated with course credit or sweets.

#### Procedure

Upon arrival at the laboratory, participants were seated at individual tables and received further instructions on screen. Specifically, they learned that they were to complete a reaction time task (i.e., the PEP-based gender stereotype assessment) followed by a questionnaire (i.e., the questionnaire-based gender stereotype assessment) and a concluding evaluation task (i.e., the selection of applicants in the hiring scenario). Participants were encouraged to contact the experimenter should questions arise. Detailed instructions were given immediately before each part of the experiment.

##### Assessment of Gender Stereotypes With the PEP

Presentation and timing mirrored the previous study. Building on the eight items of each the masculinity and femininity scale of the German version of the Personal Attributes Questionnaire (Spence and Helmreich, 1978; Runge et al., 1981), phrases representing typically male and female stereotypes were created. For example, item M10: “not at all competitive – very competitive” was represented by the phrase “... like competition” (original German wording: “... steht gern im Wettbewerb”). The gender typicality of a given phrase thus represents the within-subject factor *Gender Stereotype* (male, female). Each of these phrases was then paired with “Men” and “Women” representing the within-subject factor *Gender* (male, female). This yielded complete sentences such as “Men like competition” (male gender + male stereotype) or “Women have authority” (female gender + male stereotype). Identical to the previous study, the response prompt signaled the appropriate reaction, constituting the within-subject factor *Required Response* (“true,” “false”). Each sentence was shown three times in both male and female form (i.e., “Men are ...” vs. “Women are ...”) and with both the “true” and the “false” prompt, yielding a total of (2 × 8 sentences × 3 times) × 2 groups × 2 prompts = 192 experimental trials.

In line with Experiment 2, catch trials (“?? false – true ??” response prompt) required participants to evaluate whether the presented sentence contained spelling errors by pressing the “true” or “false” key. Therefore, sentences were generated in two versions, one featuring a spelling error and one without, yielding a total of (2 × 8 sentences × 2 spelling × 2 groups) = 64 stimuli for the catch trials. In order to reduce the demands on participants, catch trials consisted of a randomly drawn subset of 48 sentences for each participant. Therefore, each participant completed 192 standard + 48 catch = 240 trials.

This part of the experiment concluded with a 5-min filler break where participants listened to part of an audiobook and answered a set of corresponding comprehension questions.

##### Explicit Assessment of Gender Stereotypes

This was followed by explicit assessment of participants’ gender stereotypes. Specifically, they rated the very same 16 phrases employed in the PEP on a 7-point rating scale ranging from −3 = “typically male” to +3 “typically female.” Ratings for the individual masculinity and femininity items were averaged, resulting in separate scores for masculinity (Cronbach’s *α* = 0.56) and femininity (Cronbach’s *α* = 0.87). Ratings for both the masculinity and the femininity scale differed significantly from the midpoint of the scale: masculinity items were rated as more typically male, *M* = −0.62, *t*(47) = −5.95, *p* < 0.001, and femininity items were rated as more typically female, *M* = 1.13, *t*(47) = 8.25, *p* < 0.001. Consequently, ratings for femininity items were significantly different from ratings for masculinity items also, *t*(47) = 8.95, *p* < 0.001. In order to facilitate interpretation of subsequent analyses, scores for the masculinity scale were reversed, such that for both the masculinity and the femininity scale, higher values indicate more pronounced gender stereotypes. Because scores for male and female stereotypes were positively correlated (*r* = 0.3, *p* = 0.038, 95% CI: 0.02–0.54, BF_10_ = 2.26), ratings were averaged to form a global indicator of gender stereotypes.

#### Filler Task

After completing the PEP- and questionnaire-based measures, participants listened to a 5-min part from an audiobook (Harry Potter and the Philosopher’s Stone) and answered a set of comprehension questions.

#### Hiring Scenario

The study concluded with a hiring scenario, asking participants to select candidates for a total of six job descriptions (graphics designer, medical resident, teacher, lawyer, office clerk, optician) with half of them emphasizing male (agentic)^8^ and the other half emphasizing female (communal)^9^ traits.

First, for each of the six job descriptions, the job offer was displayed on screen. Second, for each job, CV’s of a set of four applicants (50% male) whose qualifications were in fact equivalent (i.e., all applicants for the graphics design job posting had bachelor and master’s degrees in the field, but from different universities) were shown. Applications differed in aspects irrelevant to the job requirements, i.e., in the type of hobbies participants listed (e.g., soccer vs. skiing vs. dancing). These CV’s were shown on screen one at a time and participants were free to go back and forth between them using on-screen buttons. After studying the CV’s, participants selected the most appropriate applicant via mouse click and continued to the next job offer. Hiring discrimination was operationalized by computing the relative frequency of *gender congruent choices*, i.e., how often participants selected one of the male applicants for a stereotypically male, or one of the female applicants for the stereotypically female job.

### Results

Prior to analyses, trials with incorrect responses (7%), as well as excessively long or short reaction times (150 < RT < 2,500 ms), were removed (0.6%). To further reduce the impact of outliers, reaction times exceeding the mean of an individual’s respective reaction time distribution^3^ by more than two SDs (3%) were deleted. Two participants were excluded from the sample because they achieved less than 80% accuracy in the PEP, resulting in a final sample of *N* = 46 (4% attrition rate).

#### Measurement of Gender Stereotypes With the PEP

**Figure 4:**
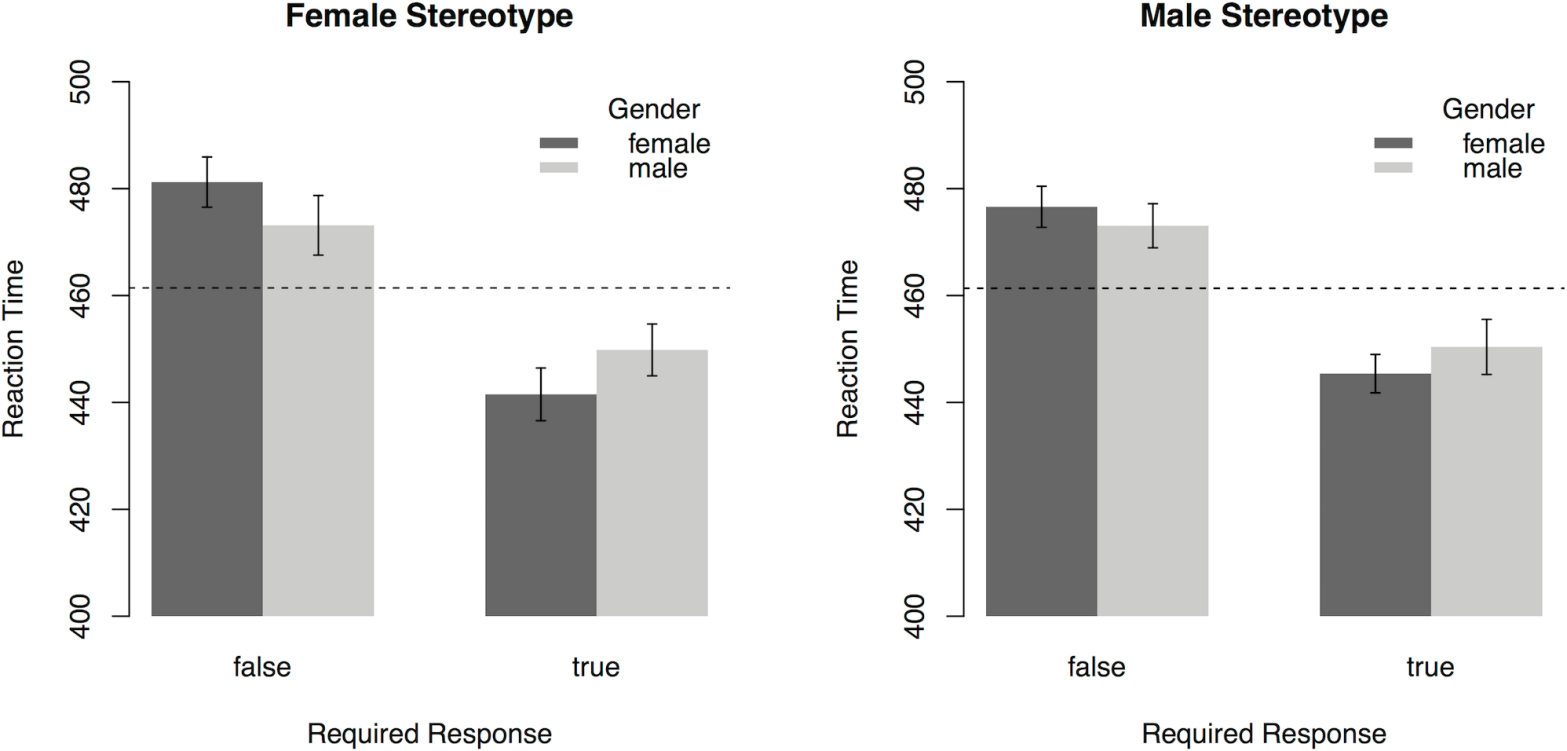
Reaction times (error bars indicate 95% CI) in the PEP depending on *Gender* named in sentence, *Required Response*, and the type of *Gender Stereotype* (Experiment 3). Dashed lines represent mean reaction time. On the sample level, there is no indication that participants associate men (women) more with gender congruent male (female) stereotypes.

To analyze general trends in gender stereotypes, participants’ reaction time for categorization of the response prompt was subjected to a 2 (*Gender Stereotype*: male, female) × 2 (*Gender*: male, female) × 2 (*Required Response*: true, false) ANOVA with repeated measurement on all factors. In addition to a main effect of *Required Response*, *F*(1, 45) = 112.41, *p* < 0.001, $\eta_{p}^{2}$ = 0.71, an interaction effect of *Required Response* × *Gender* was found, *F*(1, 45) = 13.95, *p* = 0.003, $\eta_{p}^{2}$ = 0.24. The three-way interaction of *Required Response* × *Gender* × *Gender Stereotype* fell short of significance, *F*(1, 45) = 3.48, *p* = 0.07, $\eta_{p}^{2}$ = 0.07 (see Figure 4). Thus, there was no strong evidence for the endorsement of gender stereotypes in this task on the sample level. All other effects were not significant (all *p*’s > 0.24).

#### PEP Versus Questionnaire-Based Gender Stereotypes

Similar to the procedure in the previous experiments, participants’ endorsement of gender stereotypes in the PEP is reflected by an interaction effect. Due to the fact that this iteration of the PEP assessed evaluation of stereotypic attributes in relation to two different groups (vs. evaluation of stereotypic attributes in relation to the one group of refugees), a new variable representing the interaction effect of *Gender* × *Required Response* from the ANOVA was computed separately for each participant and *Gender Stereotype* scale, such that higher values indicate more pronounced gender stereotypes. Participants’ general endorsement of gender stereotypes is thus represented by the sum of the PEP scores for the male and female stereotypes. Technically, this variable corresponds to the three-way interaction of *Gender* × *Required Response* × *Gender Stereotype* (RT_<gender stereotype>, <gender>, <required response>_):


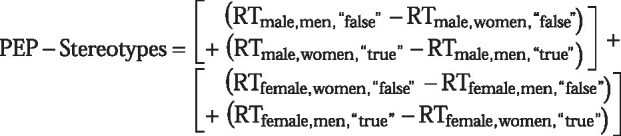


Correlating PEP-based gender stereotypes with questionnaire-based gender stereotypes revealed no significant relationship (*r* = 0.08, *p* = 0.62, 95% CI: −0.22 to 0.36, BF_10_ = 0.21).

#### Predicting Hiring Bias

To analyze the relative influence of gender stereotypes assessed by PEP and questionnaire on participants’ gender congruent choices in the hiring scenario, relative frequency of gender congruent hiring decisions was regressed on both gender stereotype scores in a multiple regression. This revealed that neither PEP-based nor questionnaire-based aggregate gender stereotypes were related to gender congruent hiring decisions (PEP: *β* = 0.21, *p* = 0.16; Questionnaire: *β* = 0.09, *p* = 0.55). Simple correlations revealed virtually identical results (PEP: *r* = 0.22, *p* = 0.15, 95% CI: −0.078 to 0.48, BF_10_ = 0.51; Questionnaire: *r* = 0.11, *p* = 0.48, 95% CI: −0.19 to 0.38, BF_10_ = 0.23).

Due to the fact that (in contrast to the questionnaire-based gender stereotypes) PEP-based stereotypes were not positively correlated^10^, the multiple regression was repeated using separate variables for male and female PEP-based gender stereotypes. As illustrated in Table 4, neither questionnaire-based nor male PEP gender stereotypes were related to gender congruent hiring (all *p*s > 0.79). In contrast, results for female PEP gender stereotypes suggested a possible relationship. Note though, that the effect fell short of significance (*p* = 0.05).

**Table 4:** Multiple regressions of gender congruent hiring decisions on PEP- and questionnaire-based gender stereotypes in Experiment 3.

Predictor	*B*	SE *B*	*β*	SE *β*	*t*	*p*
Intercept	0.45	0.04	0.49	0.03	19.67	<0.001***
PEP_male_	−0.0003	0.0013	−0.008	0.03	−0.27	0.79
PEP_female_	0.0018	0.0009	0.06	0.03	1.99	0.05
QNR	0.005	0.02	0.006	0.03	0.24	0.81

*t for standardized beta’s*.

*** *p < 0.001*.

#### Reliability of the PEP

In contrast to the previous experiments, split-half (odd-even) reliability of the PEP scores for both male and female stereotypes was low (*r* < 0.1, see also Footnote 10).

### Discussion

Extending the application of the PEP beyond the domain of racism into the realm of hiring discrimination yielded only tentative support for the incremental validity of the PEP. In contrast to findings from Experiment 2 – demonstrating that anti-immigrant behavior was predicted by PEP-based racism – individuals’ *overall* gender stereotypes as assessed with the PEP did not predict decisions in a hiring scenario. However, follow-up analyses assessing the relationship of PEP-based male and female gender stereotypes separately suggest a possible positive influence of PEP-based female gender stereotypes on hiring behavior (such that more pronounced female gender stereotypes may be related to more gender congruent hiring decisions). In addition, in contrast to the previous experiments, the PEP’s reliability in assessing gender stereotypes was unusually low. This might be due to two reasons: first, the current iteration of the PEP employed a substantially higher number of trials than those used in the previous experiments (240 trials vs. 132/204 trials), thus being more demanding for participants. Second, in order to offset an even higher number of trials due to the assessment of two gender stereotypes in relation to two genders (vs. assessment of racism toward a single social group), it was also the PEP with the lowest proportion of catch trials (20% vs. 22/33%). Recall that catch trials were included to ensure that presented sentences are actually read by participants – a prerequisite for meaningful PEP effects to emerge. Even though we do not know to date what represents the minimum viable proportion of catch trials, it appears conceivable that the 20% employed in the current PEP might have been below tolerable limits.

## General Discussion

We aimed to introduce and validate an alternative measure for the assessment of complex beliefs. Building on established paradigms such as the IRAP (Barnes-Holmes et al., 2010) and the RRT (De Houwer et al., 2015), the Propositional Evaluation Paradigm (PEP) aims to alleviate limitations inherent in their block-based design.

First and foremost, the PEP may be a useful tool to assess a *variety of beliefs* from different contexts in *a single PEP session*, because it does not rely on belief-specific instructions. Second, by eliminating the block structure of previous implicit measures (instructions and response assignments are the same for all trials, i.e., to press the key indicated by the response prompt), PEP scores are no longer susceptible to method variance due to the block factor. Finally, recall that the RRT’s block-based nature does not allow for interpretation of the overall RRT score. As discussed in detail by (De Houwer et al., 2015, p. 4), this is due to the fact that response latencies on the second block are simultaneously influenced by practice, fatigue, and response reversal effects – in addition to the effect of the belief to be measured. In contrast, the very absence of such a block structure renders the PEP immune to these effects. Importantly, these advantages do not appear to be tied to drawbacks in reliability or time required for administration: Reliability in Experiments 1 and 2 was comparable to the RRT. In contrast, the lower reliability of Experiment 3 is likely caused by an increased PEP duration combined with a comparatively low proportion of catch trials – highlighting the need to carefully consider such aspects in future research. Nevertheless, attrition rates were low in all experiments.

Experiment 1 provided first evidence for the utility of the PEP by demonstrating medium-sized^11^, significant correlations with criterion variables such as questionnaire ratings for racism, behavioral intentions indicative of racism, and political orientation (cf. De Houwer et al., 2009, on validation of implicit measures). Taken together, this suggests that the PEP may be indeed suitable for the assessment of individual differences in beliefs. Experiment 2 served to highlight the PEP’s sensitivity for indirectly assessing differences in individual beliefs – even in the absence of any explicit truth evaluation instructions during the entire task – and provided first initial evidence for the PEP’s ability to predict spontaneous behavior over and above questionnaire-based measures. Finally, Experiment 3 suggests that the PEP might have merit beyond the assessment of racism by providing tentative evidence for the predictive utility of PEP-based female gender stereotypes in predicting hiring discrimination.

### Usage Recommendations for the PEP

In the current study, we assessed either participants’ beliefs concerning both positive and negative statements about refugees (see Figure 2) or participants’ beliefs concerning the gender typicality of different traits and behaviors. This provides the advantage that differences in participants’ general reaction time for pressing the “true” compared to the “false” key (i.e., the main effect of the required response – irrespective of the presented statement) are orthogonal to the resulting effects. Even though it is entirely possible to design a PEP with just one class of statements (i.e., either all endorsing or all contradicting a certain belief), by doing so one loses the opportunity to control for reaction time differences between response keys. Although one could balance response key assignment across participants, differences in a specific participant’s key specific response speed (e.g., due to handedness) will then introduce additional error variance at the level of the individual. We therefore recommend the use of materials including both congruent and incongruent statements with regard to a specific belief.

The specific implementation of the PEP’s catch trials allows tailoring the PEP to different applications. The PEP allows assessment of individual differences in spontaneous evaluation of statements when catch trials forcing evaluation of select trials are used (Experiment 1). Including explicit truth evaluation trials into the PEP as an additional task might render the task open to influences of explicit beliefs that might become activated during the task when explicit truth evaluations are required, as was indicated by substantial positive correlations between PEP effect scores and explicit attitudes in Experiment 1. A more subtle assessment of implicit attitudes free from such an influence may be possible with variants of the PEP that use additional tasks unrelated to truth evaluation (Experiments 2 and 3: participants had to indicate whether the sentence contained a spelling error). Note that in comparison to the truth evaluation task, the orthographical judgment task might lead to a reduced reliability of the PEP scores, and to patterns of responding that deviate from what is obtained with explicit, questionnaire-based measures. Furthermore, this version of the PEP necessitates the inclusion of incorrectly spelled items, thus increasing the number of trials. Of course, running a PEP without catch trials is an option; however, this comes at the risk that participants do not process the presented sentences at all. How likely participants are to ignore the meaning of the sentences altogether (in the absence of catch trials) may be dependent on the exact nature of the employed items (e.g., this may depend on the complexity and personal relevance of the statements). However, these issues constitute open questions that await investigation in future studies.

### The PEP as an Implicit Measure

The current paper introduced the PEP as a paradigm allowing indirect assessment of participants’ endorsed beliefs. Can it also be considered to be an implicit measure? Following Moors and De Houwer (2012), an implicit measure should assess the construct of interest under conditions of automaticity. That an automatic process requires little time is among the signature features of automaticity. Sure enough, participants’ reactions on the PEP can be considered as fast (approx. 500 ms, see Figures 2, 3). Importantly, even though participants responded quickly to the probe stimuli, the PEP proved to be a valid measure of their beliefs as indicated by correlations with criterion variables. Second, the influence of participants’ evaluation of the presented statements on their reaction required by the PEP’s response prompt qualifies as *unintentional.* This is due to the fact that the presence of the response prompt “true” or “false” unequivocally determines the appropriate reaction whereas the presented sentence primes are irrelevant with regard to the to-be-executed response. Still, we concede that the findings that we obtained with the PEP might be goal-dependent in that they might vary depending on the additional task that is used in combination with the critical trials. In particular, the additional truth evaluation task that was used in Experiment 1 might have induced an “evaluative mindset” also during the critical trials of the PEP. Although such an explanation is much less likely for Experiments 2 and 3 that used a spell checking task in the additional trials, it still cannot be fully ruled out that this task might have contributed to the findings. For the time being, we thus consider the PEP to tap into conditionally automatic processes. Future studies have to investigate the automaticity conditions more systematically in order to decide which aspects of the PEP can be considered as being fully implicit.

### Outlook

To summarize, the current findings suggest that the PEP has the potential to offer a valid and reliable alternative for the assessment of individual differences in beliefs. Among its advantages are (1) easy implementation and instruction, (2) assessment of multiple beliefs in a single test session, (3) low attrition rate, and (4) easy application. This provides researchers with a promising alternative in assessing not only implicit associations but spontaneous evaluations of complex propositional statements.

## Data Availability Statement

The datasets generated for this study are available on request to the corresponding author.

## Ethics Statement

An ethics approval was not required as per applicable institutional and national guidelines and regulations because no cover-story or otherwise misleading or suggestive information was conveyed to participants (this procedure is in accordance with the ethical standards at the Institute of Psychology of the University of Jena).

## Author Contributions

FM designed, conducted, and analyzed studies and wrote the initial draft. KR designed the studies and collaborated on manuscript. Both authors contributed to manuscript revision, read, and approved the submitted version.

### Conflict of Interest

The authors declare that the research was conducted in the absence of any commercial or financial relationships that could be construed as a potential conflict of interest.

## Footnotes

^1^Whereas some authors distinguish implicit from explicit measures based on participants’ awareness of the measurement, others argue that implicit measures are assessing different constructs than explicit measures. In contrast, (Gawronski and De Houwer, 2014, p. 3) argue “that it is conceptually more appropriate to classify different measures in terms of whether the to-be-measured psychological attribute influences participants’ responses on the task in an automatic fashion.”

^2^Sample size was chosen to allow detection of medium-sized correlations between PEP-based racism scores and criterion variables as have been reported in previous research (De Houwer et al., 2015) with a power of at least 0.80.

^3^Applies also to Experiment 2 and 3.

^4^Separate distributions were employed for each combination of participant and trial type.

^5^Recall that PEP scores are derived from the interaction of the evaluation of the presented belief (i.e., sentence) and the classification of the response prompt. Failing to classify the response prompt correctly would thus compromise resulting PEP scores. Hence, we opted to exclude those participants.

^6^Based on the correlation between PEP-based racism and behavioral intentions in Experiment 1 (*r* = −0.33) a sample size of at least *N* = 52 is needed to detect a relationship of similar direction and size with a power of 0.8 at an alpha level of 0.05.

^7^Sample size was based on the relationship between PEP-based racism and actual behavior from Experiment 2, necessitating a sample size of *N* = 48 to achieve a power of 0.8 at an alpha level of 0.05.

^8^Example: “Garrelts-Optics is a rapidly growing and dynamic company - always on the lookout for enthusiastic employees. The position requires a strong personality and the ability for decision making. In addition the candidate should be able to work independently and take on managerial responsibilities.”

^9^Example: “Kirsch is a renowned marketing agency. Currently they are looking for another graphics designer to handle the growing number of clients. The position requires someone who is cooperative, caring and attentive to clients’ needs, and open for new ideas.”

^10^PEP scores for the male and female stereotypes were in fact correlated negatively (*r* = −0.45, *p* = 0.002). Whereas unexpected, this is most likely due to two factors. First, as elaborated previously, the influence of the experimental factors *Gender*, *Required Response*, and *Gender Stereotype* on reaction times in the PEP was characterized by a two-way interaction of *Required Response* and *Gender*. Second, reaction times characterized by this interaction enter the formula for the calculation of the respective PEP effects for male and female stereotypes with opposite signs, thus driving the negative correlation.

^11^See (Field, 2009, p. 57) for classification of effect sizes.
